# ISA-2011B, a Phosphatidylinositol 4-Phosphate 5-Kinase α Inhibitor, Impairs CD28-Dependent Costimulatory and Pro-inflammatory Signals in Human T Lymphocytes

**DOI:** 10.3389/fimmu.2017.00502

**Published:** 2017-04-26

**Authors:** Martina Kunkl, Nicla Porciello, Marta Mastrogiovanni, Cristina Capuano, Federica Lucantoni, Chiara Moretti, Jenny L. Persson, Ricciarda Galandrini, Raffaella Buzzetti, Loretta Tuosto

**Affiliations:** ^1^Department of Biology and Biotechnology Charles Darwin, Sapienza University, Laboratory Affiliated to Istituto Pasteur Italia-Fondazione Cenci Bolognetti, Rome, Italy; ^2^Department of Experimental Medicine, Sapienza University, Rome, Italy; ^3^Division of Experimental Cancer Research, Department of Laboratory Medicine, Clinical Research Center, Lund University, Malmö, Sweden; ^4^Department of Molecular Biology, Umeå University, Umeå, Sweden

**Keywords:** CD28 co-stimulation, PIP5K, T lymphocytes, pro-inflammatory cytokines, T1D

## Abstract

Phosphatidylinositol 4,5-biphosphate (PIP2) is a membrane phospholipid that controls the activity of several proteins regulating cytoskeleton reorganization, cytokine gene expression, T cell survival, proliferation, and differentiation. Phosphatidylinositol 4-phosphate 5-kinases (PIP5Ks) are the main enzymes involved in PIP2 biosynthesis by phosphorylating phosphatidylinositol 4-monophosphate (PI4P) at the D5 position of the inositol ring. In human T lymphocytes, we recently found that CD28 costimulatory molecule is pivotal for PIP2 turnover by recruiting and activating PIP5Kα. We also found that PIP5Kα is the main regulator of both CD28 costimulatory signals integrating those delivered by TCR as well as CD28 autonomous signals regulating the expression of pro-inflammatory genes. Given emerging studies linking alterations of PIP2 metabolism to immune-based diseases, PIP5Kα may represent a promising target to modulate immunity and inflammation. Herewith, we characterized a recently discovered inhibitor of PIP5Kα, ISA-2011B, for its inhibitory effects on T lymphocyte functions. We found that the inhibition of PIP5Kα lipid-kinase activity by ISA-2011B significantly impaired CD28 costimulatory signals necessary for TCR-mediated Ca^2+^ influx, NF-AT transcriptional activity, and IL-2 gene expression as well as CD28 autonomous signals regulating the activation of NF-κB and the transcription of pro-inflammatory cytokine and chemokine genes. Moreover, our data on the inhibitory effects of ISA-2011B on CD28-mediated upregulation of inflammatory cytokines related to Th17 cell phenotype in type 1 diabetes patients suggest ISA-2011B as a promising anti-inflammatory drug.

## Introduction

Phosphatidylinositol 4,5-biphosphate kinases (PIP5Ks) are a family of lipid kinases that phosphorylate phosphatidylinositol 4-phosphate on the D5 position of the inositol ring, thus inducing the neo-synthesis of phosphatidylinositol 4,5-biphosphate (PIP2) (1). In humans, PIP5K family consists of three isoforms (α, β, and γ) and further splice variants. T lymphocytes express all PIP5K isoforms, each displaying a specific subcellular localization that ensures the spatiotemporal organization of distinct PIP2 pools (2–5).

In T lymphocytes, PIP2 is a precursor for second messengers, inositol triphosphate (IP3), and diacylglycerol (DAG). DAG is mainly involved in the activation of the RAS/protein kinase C θ pathway (6, 7), whereas soluble IP3 mediates the release of Ca^2+^ from the intracellular stores, an event that induces the activation of the Ca^2+^/calmodulin-dependent calcineurin. Calcineurin is responsible for the dephosphorylation and activation of NF-AT transcription factors, which translocate into the nucleus and contribute to the transcriptional activation of pivotal genes, such as the IL-2 gene (8). This pivotal signaling pathway requires signals delivered by both TCR and CD28 co-stimulatory molecule (9, 10).

Phosphatidylinositol 4,5-biphosphate serves also as a substrate of class 1 PI3K that, by phosphorylating PIP2 on the D3 position of the inositol ring, generates phosphatidylinositol 3,4,5-triphosphate (PIP3) lipids (11). PIP3 lipids recruit and activate the phosphoinositide-dependent protein kinase 1 (PDK1)/Akt pathway. PI3K-PDK1/Akt has been involved in CD28 signals regulating the activation of the transcription factor NF-κB (12–15). NF-κB signaling pathway selectively regulates the expression of several genes, including survival genes (16) and pro-inflammatory cytokine/chemokine (17, 18). Moreover, PI3K/PDK1/Akt also activates the mammalian target of rapamycin complex 1 (mTORC1) that is an important regulator of glucose metabolism (19). In particular, by upregulating the glycolytic pathway, PI3K/PDK1/Akt/mTORC1 favors the differentiation of specific inflammatory Th cell subsets, such as Th17 (20), which are involved in the pathogenesis of several autoimmune diseases (21).

Among all PIP5K isoforms, PIP5Kα is predominantly responsible for PIP2 synthesis in response to TCR and CD28 co-engagement. In particular, we have recently demonstrated that CD28 recruits and activates PIP5Kα at the immunological synapse (IS), thus inducing the PIP2 levels necessary for sustaining TCR-stimulated Ca^2+^ influx, NF-AT nuclear translocation, and IL-2 gene expression (3, 4). We also found that PIP5Kα is the main regulator of CD28 autonomous signals that induce NF-κB transcriptional activity and the transcription of pro-inflammatory genes (4), in a PI3K-dependent manner (17). Given the pivotal role of PIP5Kα in the regulation of PIP2 synthesis in T lymphocytes and emerging studies, which link alterations of PIP2 metabolism to immune-based diseases (22), PIP5Kα may represent a promising target to modulate immunity and inflammation.

A small molecule derivative of 1,2,3,4-tetrahydroisoquinoline ISA-2011B has been recently identified as a potential inhibitory drug of PIP5Kα. ISA-2011B was found to bind with high affinity to PIP5Kα and to inhibit its protein expression to suppress growth of prostate tumor in mouse models (23, 24). In this study, we further characterized the mechanisms of action of ISA-2011B and the effects of this agent on T lymphocyte activation and pro-inflammatory functions. We found that ISA-2011B was efficient in inhibiting PIP5Kα lipid-kinase activity in T lymphocytes, without affecting PIP5Kα protein expression. We also evidenced that PIP5Kα activity inhibition by ISA-2011B strongly impairs CD3/CD28-induced Ca^2+^ influx, NF-AT transcriptional activity, and IL-2 gene expression as well as CD28 autonomous signals regulating NF-κB transcriptional activation and pro-inflammatory cytokine/chemokine gene expression. Finally, the results obtained by analyzing the effects of ISA-2011B on CD28-mediated upregulation of inflammatory IL-8, IL-6, and IL-17A genes in T lymphocytes from type 1 diabetes (T1D) patients suggest ISA-2011B as a promising anti-inflammatory drug.

## Materials and Methods

### T1D Patients and Controls

Fourteen patients (male/female ratio = 1) with a clinically defined T1D, in accordance with the guidelines of the Expert Committee on the Diagnosis and Classification of Diabetes Mellitus (25), were enrolled from the Diabetes Unit of S.M. Goretti Hospital, Polo Pontino (Sapienza University of Rome). The Ethical Committee of the S.M. Goretti Hospital has approved the Study and all individuals gave their informed consent in written form. Patients were between 18 and 63 years old, had disease duration from 1 to 31 years, and glycated hemoglobin (HbA1c)% from 6.2 to 12.4. None of the patients showed any evidence of micro or macroangiopathy (Table 1). Fifteen healthy donors (HD) buffy coats from the blood bank of Sapienza University (Rome, Italy) with no previous history of autoimmune diseases were used as controls.

**Table 1:** **General characteristics of patients with type 1 diabetes (T1D) including the phenotype of peripheral blood T lymphocytes**.

Number of T1D patients	Age (years)	Diabetes duration (years)	% HbA1c	CD4/CD8 ratio	% CD28+ cells
14	34.4 ± 14	13.9 ± 9.8	8 ± 1.4	1.9 ± 0.8	87 ± 7

*Values indicate mean ± SD. HbA1c, glycated hemoglobin*.

### Cells, Abs, and Reagents

Human primary T cells were enriched from PBMCs of HD or T1D patients and cultured in RPMI 1640 supplemented with 5% human serum (Euroclone, UK), l-glutamine, penicillin, and streptomycin. CH7C17 Jurkat T cell line expressing CD28WT has been previously described (26). Murine L cells transfected with human, HLA-DRB1*0101 (5-3.1), and 5-3.1 co-transfected with B7.1/CD80 (5-3.1/B7) have been previously described (10, 27). The following Abs were used: goat anti-PIP5Kα (N-20), goat anti-PIP5Kα (C17), and mouse anti-HA (Santa Cruz Biotechnology, CA, USA); mouse anti-CD28.2, mouse anti-CD3 (UCHT1), and goat anti-mouse (GAM) (BD Biosciences, Milan, Italy). PI4,5P2, PI4P, PMA, and A23871 were purchased from Sigma-Aldrich (Milan, Italy) and SEB were from Toxin Technology (Sarasota, FL, USA). ISA-2011B has been previously described (24). PIP5K small molecule inhibitor was from Cancer Research Technology (CRT, London, UK).

### Plasmids, Cell Transfection, and Luciferase Assays

HA-tagged PIP5Kα was previously described (4). The NF-κB luciferase gene under the control of six thymidine kinase NF-κB sites was kindly provided by J. F. Peyron (Faculté de Médicine Pasteur, Nice, France). The NF-AT luciferase reporter construct containing the luciferase gene under the control of the human IL-2 promoter NF-AT-binding site was kindly provided by C. Baldari (University of Siena, Siena, Italy).

For luciferase assays, 10^7^ Jurkat cells were electroporated (at 260 V, 960 μF) in 0.5 ml RPMI 1640 supplemented with 20% FCS with 2 µg NF-κB luciferase or 10 µg NF-AT luciferase together with 5 µg pEGFP, keeping the total amount of DNA constant (40 µg) with empty vector. Twenty-four hours after transfection, cells were stimulated with 5-3.1 or 5-3.1/B7 cells pre-pulsed or not with SEB (1 µg/ml) at 37°C for 6 h in the presence of DMSO, as a vehicle control, or the indicated concentration of ISA-2011B. Luciferase activity was measured according to the manufacturer’s instruction (Promega). Luciferase activity determined in triplicates was expressed as arbitrary luciferase units after normalization to GFP values.

### PIP5Kα Kinase Assays and Immunoblotting

Primary T cells (20 × 10^6^) were pre-incubated for 6 h with the indicated concentration of ISA-2011B or DMSO, stimulated for 5 min with anti-CD28 (5 µg/ml), or anti-CD3 (5 µg/ml) Abs crosslinked with 20 µg/ml of GAM. CH7C17 CD28WT Jurkat cells were transfected with HA-PIP5Kα WT and treated for 6 h with the indicated concentration of ISA-2011B or DMSO. Cells were lysed for 30 min on ice in 1% Non-idet P-40 lysis buffer in the presence of inhibitors of proteases and phosphatases. Extracts were precleared for 1 h with Protein-G Sepharose, endogenous or exogenous PIP5Kα were immunoprecipitated and incubated with PI4P as previously described (28). Briefly, PI4P was resuspended in assay buffer (30 mM HEPES pH 7.4, 100 mM KCl, 1 mM EGTA, 2 mM MgCl_2_, 0.05% NP-40) and sonicated. Immunoprecipitated PIP5Kα was incubated with 50 µl assay buffer containing PI4P, MgATP (50 µM), and ^32^γ-ATP (adenosine triphosphate) for 15 min at 30°C. The organic phase that contained PIP2 was separated by thin-layer chromatography (TLC) on Silica gel 20 × 10 plates (Merck, Darmstadt, Germany). The radioactive lipids were visualized by autoradiography. The identity of PIP2 and PI4P was confirmed by comparison with standard phospholipids revealed by iodine vapor. The spot corresponding to PIP2 was quantified by densitometric analysis using the ImageJ program (National Institute of Health, USA). Primary T cells were stimulated as indicated at 37°C. At the end of incubation, cells were harvested and lysed for 30 min on ice in 1% Non-idet P-40 lysis buffer in the presence of inhibitors of proteases and phosphatases. Extracts were resolved by SDS-PAGE and blotted onto nitrocellulose membranes. Blots were incubated with the indicated primary antibodies, extensively washed, and after incubation with horseradish peroxidase (HRP)-labeled goat anti-rabbit or HRP-labeled GAM (Amersham), or (HRP)-labeled donkey anti-goat Abs (Santa Cruz Biotechnology) developed with the enhanced chemiluminescence’s detection system (GE Healthcare).

### Cytotoxicity Assay

The cytotoxicity of ISA-2011B on primary T cells was evaluated by both propidium iodide (PI) and Annexin V conjugates staining by using a commercially available kit (Molecular Probes Inc., Invitrogen, UK). Primary T cells were plated at 2 × 10^6^ cells/ml in 48-well plates and treated with different doses of ISA-2011B or DMSO for 6 or 24 h. Cytotoxicity was analyzed by a BD Biosciences FACScalibur (Mountain View, CA, USA) by quantifying the percentage of PI or Annexin V-positive cells. Results were calculated from at least three independent experiments and statistically analyzed using Student’s *t*-test.

### Measurement of Intracellular Calcium Concentration

Primary T cells (1.5 × 10^6^/ml) were treated for 6 h with DMSO or 10 µM ISA-2011B, loaded with 20 µM Fluo-3 AM (Sigma) for 30 min at 37°C in 300 µl of RPMI 1640. Loaded cells were then washed and activated with anti-CD3 plus anti-CD28 Abs crosslinked with GAM at 37°C and immediately analyzed by a cytofluorimeter (FACScalibur, BD Biosciences). Changes in cell fluorescence were monitored every 24 s for 10 min by measuring fluorescence emission at 530 nm. The concentration of intracellular calcium [Ca^2+^]_i_ was calculated according to Grynkiewicz et al. (29).

### Real-time PCR

Primary T cells or Jurkat cells were stimulated for the indicated times with 2 µg/ml anti-CD28.2 or anti-CD3 Abs (UCHT1) crosslinked with 2 µg/ml GAM Abs. Total RNA was extracted using RNeasy MicroKit (Qiagen) and was reverse-transcribed into cDNA by using Moloney murine leukemia virus reverse transcriptase (Invitrogen). TaqMan Universal PCR Master Mix, IL-8, IL-6, IL-17A, IL-2, and GAPDH primer/probe sets were purchased from Applied Biosystems. The relative quantification was performed using the comparative *C*_T_ method. The mean value of primary T cells from HD stimulated with control isotype matched Abs was used as calibrator.

### Statistical Analysis

Parametrical statistical analysis (mean and SD) was performed to evaluate differences between continuous variables through Prism 5.0 (GraphPad Software, San Diego, CA, USA) using standard unpaired *t*-test. For multiple group comparisons, significant differences were calculated using the non-parametric Mann–Whitney *U* test, and linear regression analysis was performed using the Pearson chi-squared test. For all tests, *P* values < 0.05 were considered significant.

## Results

### ISA-2011B Inhibits PIP5Kα Lipid-Kinase Activity

**Figure 1:**
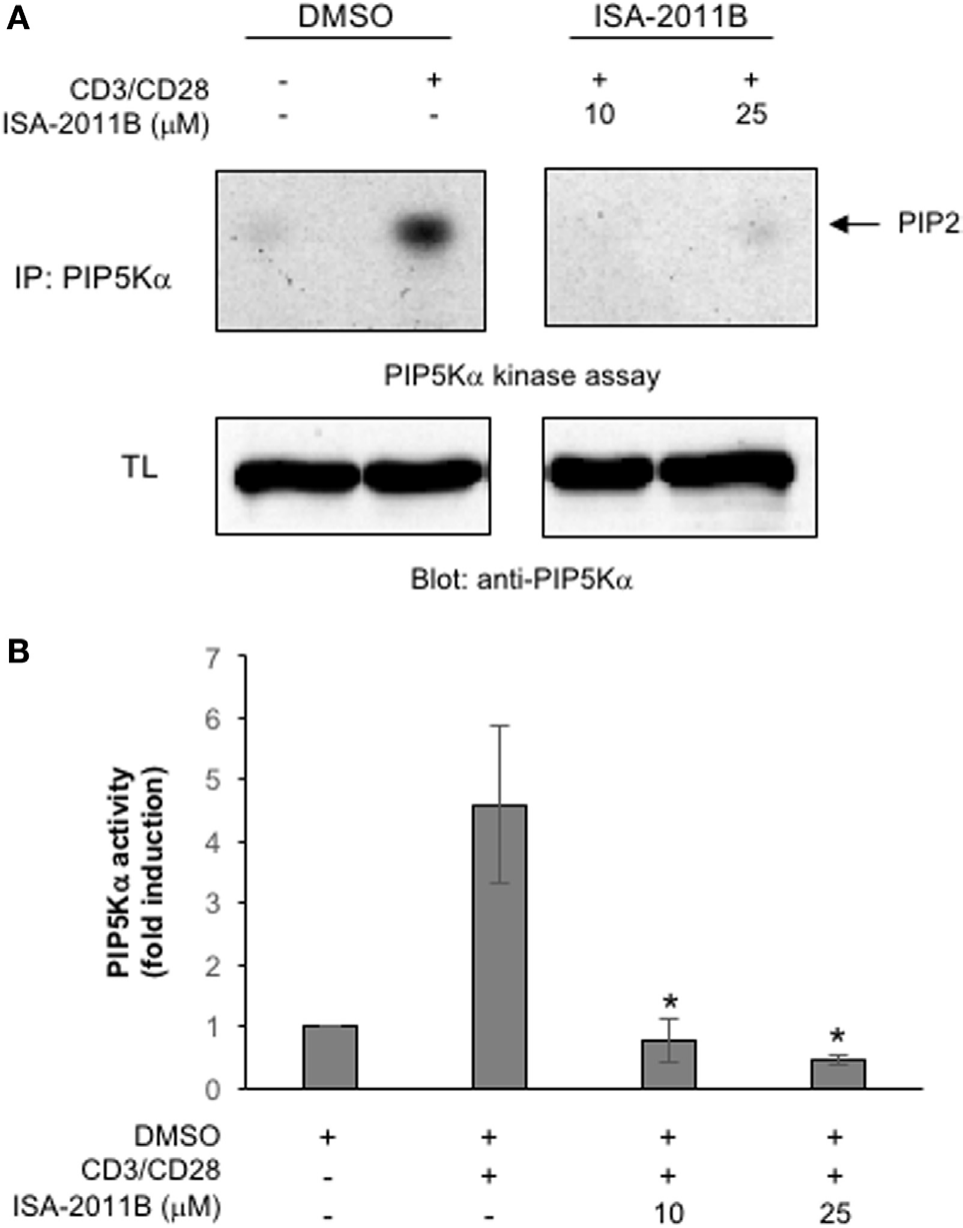
**ISA-2011B inhibits phosphatidylinositol 4-phosphate 5-kinase (PIP5K)α lipid-kinase activity in primary T cells**. **(A)** Primary T cells from healthy donors (HD) were treated for 6 h with DMSO or the indicated concentrations of ISA-2011B and then unstimulated (ctr) or stimulated for 5 min with saturating concentration of anti-CD3 (5 µg/ml) plus anti-CD28 Abs crosslinked with goat anti-mouse (20 µg/ml). PIP5K kinase assays were performed on anti-PIP5Kα immunoprecipitations and the reaction products were subjected to thin-layer chromatography followed by autoradiography (upper panel). An equal amount of cell lysate was analyzed for PIP5Kα content by Western blotting (lower panel). **(B)** Fold inductions were quantified by densitometric analysis and normalized to PIP5Kα levels. Bars show the mean ± SD of two experiments. Asterisks (*) indicate *P* < 0.05 calculated by Student’s *t*-test.

**Figure 2:**
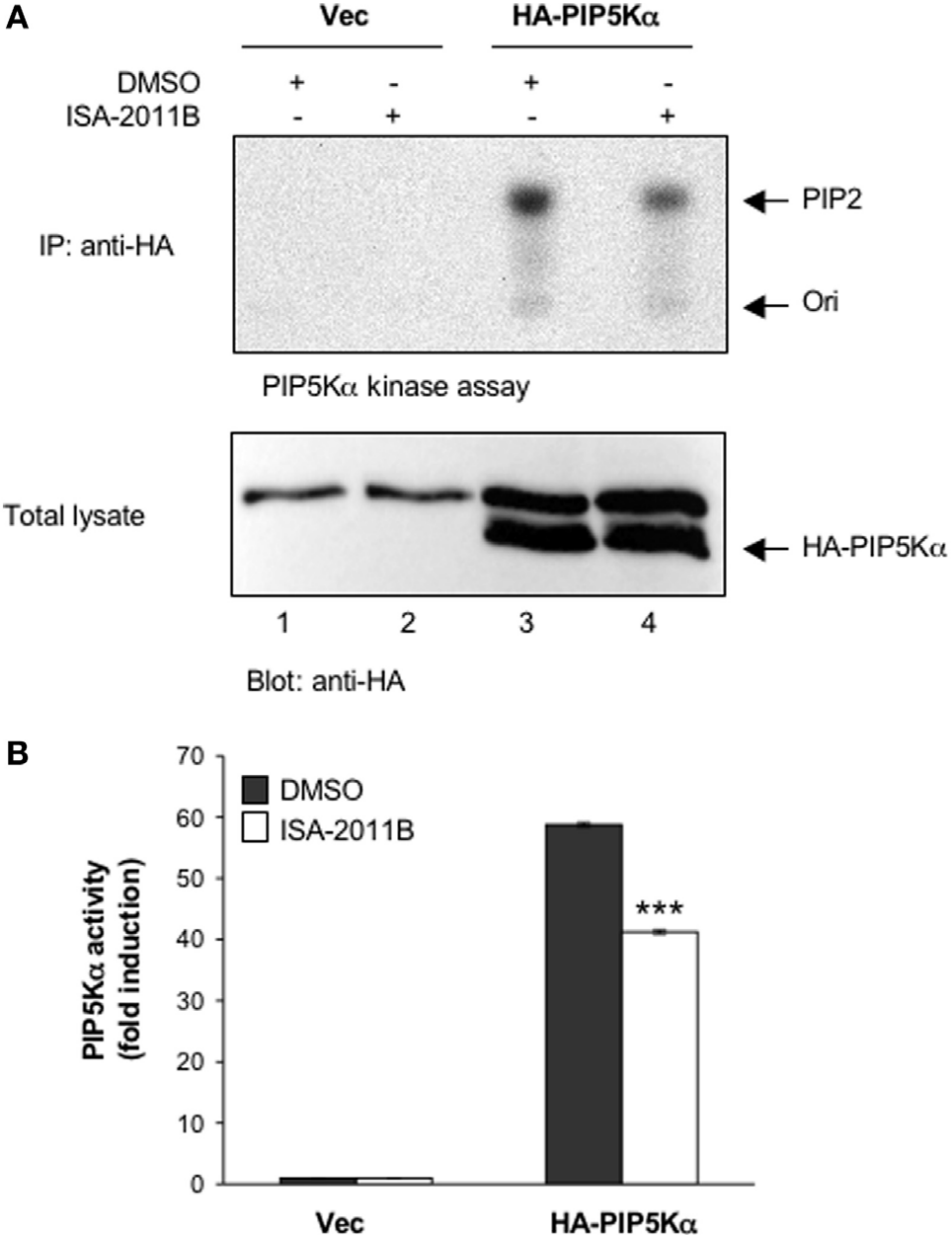
**ISA-2011B inhibits exogenously expressed PIP5Kα lipid-kinase activity**. **(A)** Jurkat cells were transfected and cultured for 24 h with 20 µg control empty vector (Vec) or 20 µg HA-PIP5Kα and then treated for further 6 h with DMSO or 25 µM ISA-2011B. PIP5Kα kinase assays performed on anti-HA IP (upper panel). HA-PIP5Kα content was analyzed by western blotting (lower panel). **(B)** Fold inductions were quantified by densitometric analysis and normalized to HA-PIP5Kα levels. Data are expressed as fold induction over the basal level of cells transfected with empty vector and treated with DMSO. Bars show the mean ± SD of two experiments. Asterisks (***) indicate *P* < 0.001 calculated by Student’s *t*-test, compared with cells transfected with HA-PIP5Kα and treated with DMSO.

ISA-2011B is a diketopiperazine fused C-1 indol-3-yl substituted 1,2,3,4-tetrahydroisoquinoline derivative that exhibits a high binding affinity to PIP5Kα and was described to inhibit PIP5Kα protein expression in prostate cancer (PC)-3 cells (24). Here we further characterized ISA-2011B functions, by analyzing its effects on the lipid-kinase activity of PIP5Kα, in primary T lymphocytes. Primary T cells were pre-treated for 6 h with the indicated concentration of ISA-2011B or DMSO, as a vehicle control, and then stimulated for 5 min with isotype matched or anti-CD3 plus CD28 Abs. Our previous data evidenced that these stimulating conditions were efficient as anti-CD3 or anti-CD28 alone in activating PIP5Kα (3). Endogenous PIP5Kα was immunoprecipitated and its lipid-kinase activity was evaluated (Figure 1A). As previously demonstrated (3), CD3 plus CD28 stimulation by agonistic Abs strongly increased the lipid-kinase activity of PIP5Kα in primary T cells. Pre-treatment of T cells with either 10 or 25 µM of ISA-2011B strongly impaired CD3/CD28-mediated induction of PIP5Kα activity (Figures 1A,B). Although to a lesser extent, similar inhibitory effects of ISA-2011B were observed on constitutive activity of exogenously expressed PIP5Kα in Jurkat cells (Figures 2A,B). For instance, we have previously demonstrated that PIP5Kα exhibits high constitutive activity when over-expressed (4). The analysis of PIP5Kα content revealed that ISA-2011B did not affect PIP5Kα protein levels after 6 h of treatment at neither 10 nor 25 µM (Figure S1 in Supplementary Material). Consistently with the data on PC-3 cells (24), treatment of primary T cells with 25 µM ISA-2011B significantly inhibited PIP5Kα protein expression after overnight and 24 h (Figure S1A in Supplementary Material).

Collectively these data evidence that ISA-2011B acts as a lipid-kinase inhibitor of PIP5Kα.

### ISA-2011B Impairs CD28 Costimulatory Signals

**Figure 3:**
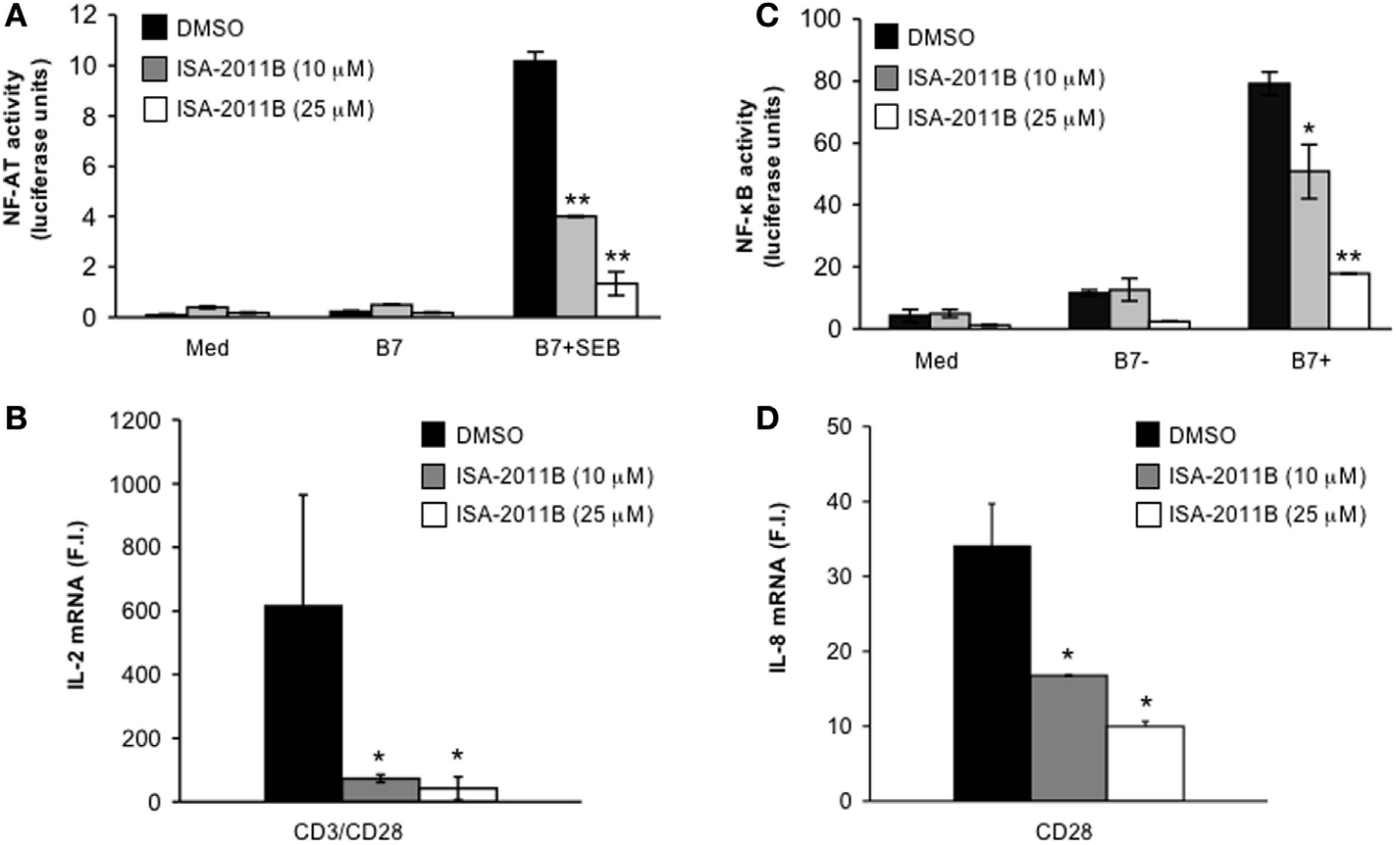
**ISA-2011B impairs CD28 costimulatory functions in Jurkat T cells**. **(A)** NF-AT luciferase activity of Jurkat cells treated for 6 h with DMSO or the indicated concentration of ISA-2011B and stimulated in the absence (med) or presence of 5-3.1/B7 (B7) cells pre-pulsed or not with 1 µg/ml SEB. The results are expressed as the mean of luciferase units ± SD after normalization to GFP values. The data are representative of three independent experiments. **(B,D)** Real-time PCR was used to measure IL-2 mRNA **(B)** and IL-8 mRNA levels **(D)** in Jurkat cells treated with DMSO or the indicated concentration of ISA-2011B and stimulated for 6 h with control IgG or anti-CD28 Abs **(D)** or anti-CD3 plus anti-CD28 Abs **(B)**. Data are expressed as fold inductions (F.I.) over the basal level of cells stimulated with control IgG and treated with DMSO. Bars show the mean ± SD of three independent experiments. **(C)** NF-κB luciferase activity of Jurkat cells treated for 6 h with DMSO or the indicated concentration of ISA-2011B and stimulated in the absence (med) or presence of B7-negative (%-3.1) or B7-positive (5-3.1/B7) cells. The results are expressed as the mean of luciferase units ± SD after normalization to GFP values. The data are representative of three independent experiments. Asterisks (*) and (**) indicate *P* < 0.05 and *P* < 0.01, respectively, calculated by Student’s *t*-test.

We have demonstrated that the lipid-kinase activity of PIP5Kα is crucial for the regulation of both CD3/CD28 costimulatory signals and CD28 unique signals, in both primary T cells and Jurkat cells (3, 4). Pre-treatment of Jurkat cells with ISA-2011B strongly impaired NF-AT transcriptional activity induced by TCR stimulation alone (Figure S2A in Supplementary Material) or TCR and CD28 co-engagement (Figure 3A). ISA-2011B treatment strongly reduced IL-2 mRNA levels induced by TCR and CD28 co-engagement (Figure 3B; Figure S2B in Supplementary Material). CD3 stimulation alone induced only a slight increase (twofolds) of IL-2 gene expression that was not significantly inhibited by ISA-2011B (Figure S2B in Supplementary Material). By contrast, ISA-2011B treatment did not exert any significant effects on NF-AT transcriptional activity induced by PMA plus A23871, which bypass PIP2 involvement (Figure S2C in Supplementary Material). CD28 autonomous signals regulating both NF-κB transcriptional activation (Figure 3C) and RelA/NF-κB-dependent IL-8 gene transcription (17, 18) were also significantly inhibited in ISA-2011B-treated Jurkat cells (Figure 3D).

Before testing the effects of ISA-2011B on primary T cell functions, we performed cell viability analyses to identify the dose of ISA-2011B with lower cytotoxic activity. The results obtained by analyzing both cytotoxic (PI incorporation) or apoptotic (Annexin V staining) activities of different ISA-2011B doses revealed a significant induction of both cell mortality (Figure S3A in Supplementary Material) and apoptosis (Figure S3B in Supplementary Material) at 25 µM. By contrast, at the dose of 10 µM, ISA-2011B failed to induce significant cell death in primary T cells from HD. Thus, 10 µM was chosen as the optimal dose for the next experiments on primary T cells.

**Figure 4:**
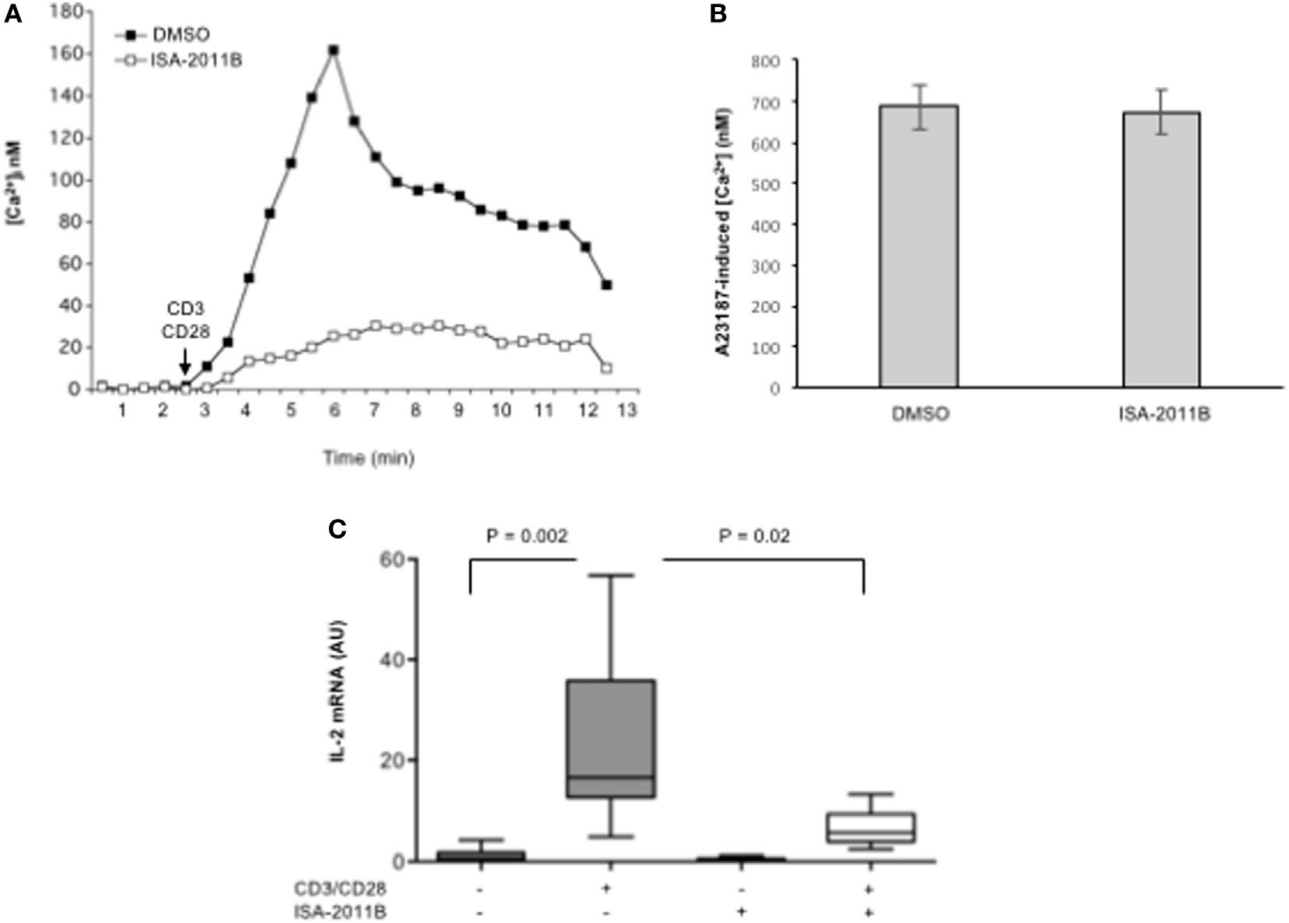
**ISA-2011B impairs TCR- and CD28-induced increase of [Ca^2+^]_i_ and IL-2 gene expression in primary T cells**. **(A,B)** Primary T cells from HD were treated for 6 h with DMSO or ISA-2011B (10 µM) and after loading T cells with Fluo-3-AM, Ca^2+^ levels ([Ca^2+^]_i_) were measured by cytofluorimetric analysis after stimulation with crosslinked anti-CD3 plus anti-CD28 Abs **(A)** or A23871 (2 µg/ml) for 10 min. Data are representative of three independent experiments. **(C)** Real-time PCR was used to measure IL-2 mRNA levels in primary T cells from healthy donors (*n* = 5) treated with DMSO or ISA-2011B (10 µg/ml) and stimulated for 6 h with crosslinked anti-CD3 plus anti-CD28 Abs. Values, normalized on GAPDH, are expressed as arbitrary units. Median values: DMSO = 0.55; DMSO CD3 plus CD28 = 16.59; ISA-2011B = 0.25: ISA-2011B CD3 plus CD28 = 5.68. Lines represent median values and *P* values are indicated where significant (Mann–Whitney).

To assess whether the inhibition of PIP5Kα lipid-kinase activity by ISA-2011B could affect early and late CD28 costimulatory signals, we analyzed both TCR/CD28-dependent Ca^2+^ signaling cascade and IL-2 gene expression in primary T cells. We have, indeed, demonstrated that CD28-co-stimulation is essential to induce a stronger and prolonged Ca^2+^ influx compared with TCR individual ligation, optimal NF-AT nuclear translocation, and IL-2 gene expression by regulating PIP2 turnover through PIP5Kα activation (3). Pre-treatment of primary T lymphocytes with 10 µM ISA-2011B strongly impaired the increment of Ca^2+^ levels mediated by CD3/CD28 co-stimulation (Figure 4A), without affecting Ca^2+^ influx induced by A23871 ionophore (Figure 4B). The inhibition rate of Ca^2+^ levels by ISA-2011B (85%) where comparable to those exerted on PIP5Kα lipid-kinase activity (82%). Consistently with these data, ISA-2011B treatment strongly inhibited IL-2 transcription induced by TCR plus CD28 stimuli (Figure 4C).

### ISA-2011B Inhibits CD28 Pro-inflammatory Functions

Most of CD28 unique signaling converges to the activation of NF-κB (30, 31) and to the expression of several genes, including pro-inflammatory cytokines and chemokines (17).

**Figure 5:**
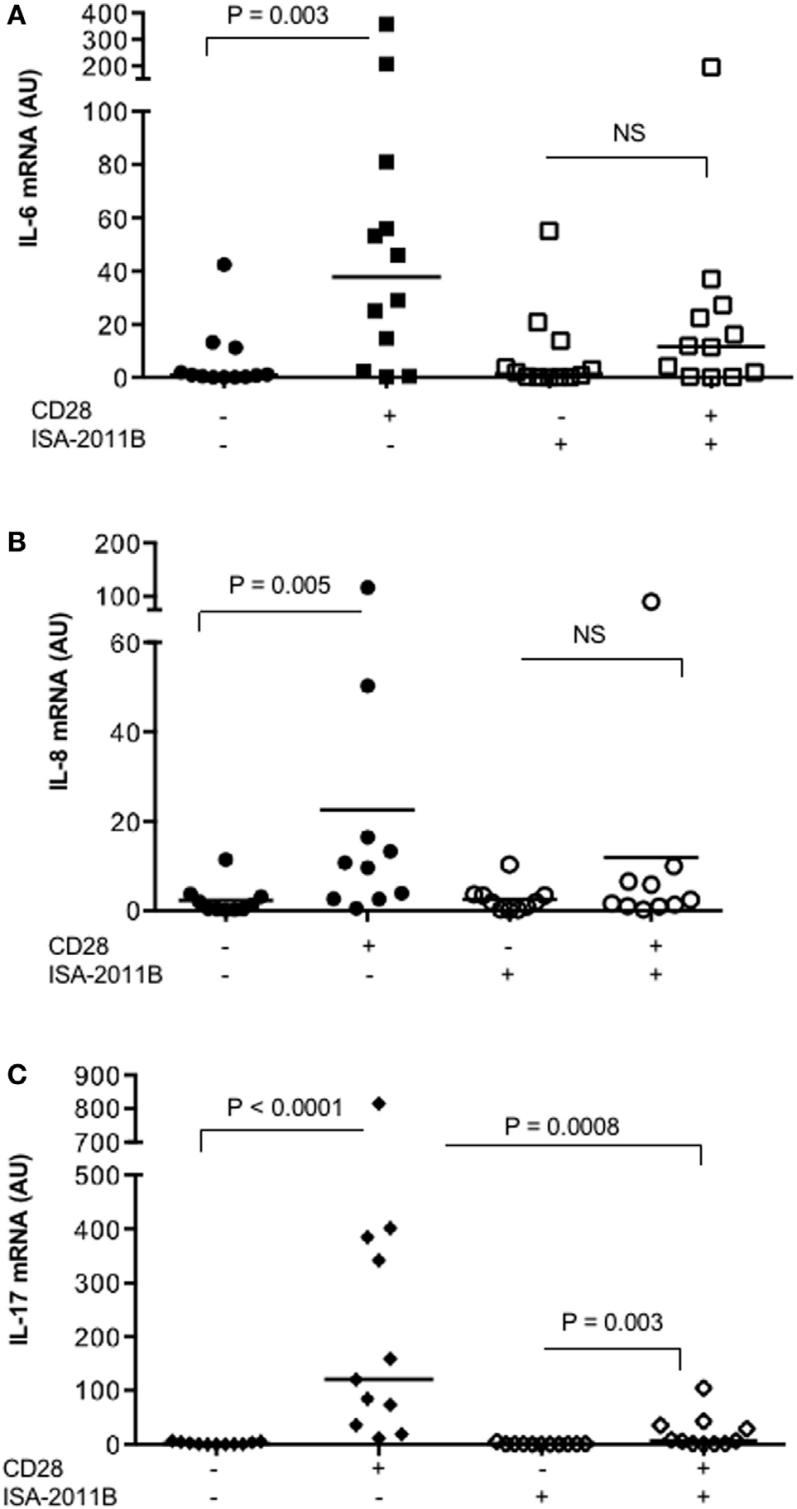
**ISA-2011B impairs CD28 autonomous signals inducing the expression of pro-inflammatory cytokine/chemokine**. Primary T cells from healthy donors subjects (*n* = 12) were treated with DMSO or ISA-2011B (10 µM) and stimulated for 6 h **(A,B)** or 24 h **(C)** with control isotype-matched mAb or crosslinked anti-CD28.2 Abs. IL-6 **(A)**, IL-8 **(B)**, and IL-17A **(C)** mRNA levels were measured by real-time PCR and values, normalized on GAPDH, expressed as arbitrary units. Median values: IL-6, DMSO = 0.89, DMSO CD28 = 37.66, ISA-2011B = 1.33, ISA-2011B CD28 = 11.66; IL-8, DMSO = 0.9, DMSO CD28 = 10.19, ISA-2011B = 1.9, ISA-2011B CD28 = 2.0; IL-17A, DMSO = 0.76, DMSO + CD28 = 120.4, ISA-2011B = 0.69, ISA-2011B CD28 = 7.0. Lines represent median values and *P* values are indicated where significant (Mann–Whitney). NS = not significant.

We next tested whether the impairment of PIP5Kα activity by ISA-2011B might affect CD28 pro-inflammatory functions in primary T cells. Consistently with our previous data (17), CD28 stimulation, in the absence of TCR engagement, significantly increased the mRNA levels of IL-6, IL-8, and IL-17A in T lymphocytes from HD (Figure 5). On the contrary, no significant increase in IL-6 and IL-8 gene expression was induced by CD28 stimulation in ISA-2011B-treated T lymphocytes compared with cells treated with DMSO, as vehicle control (Figures 5A,B). CD28-induced IL-17A cytokine expression was also strongly downregulated by 94% following ISA-2011B treatment (Figure 5C).

These data suggest ISA-2011B as a drug candidate to dampen CD28 pro-inflammatory functions in T lymphocytes.

### ISA-2011B Impairs CD28-Dependent Pro-inflammatory Cytokine Expression in T1D T Lymphocytes

**Figure 6:**
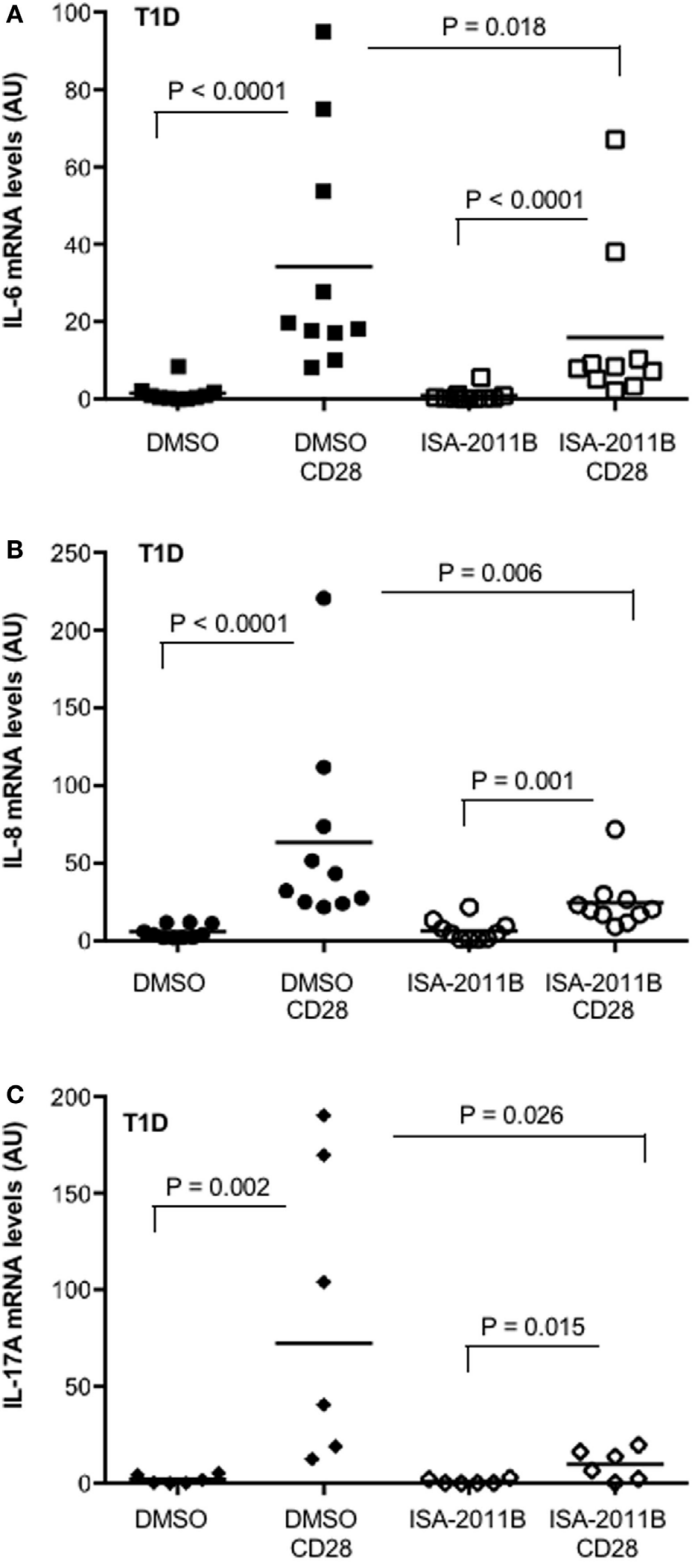
**PIP5Kα inhibition by ISA-2011B impairs CD28-mediated upregulation of pro-inflammatory cytokines in primary T cells from type 1 diabetes (T1D) patients**. **(A)** Primary T cells from T1D patients subjects were treated with DMSO or ISA-2011B (10 µg/ml) and stimulated for 6 h **(A,B)** or 24 h **(C)** with control isotype-matched mAb or crosslinked anti-CD28.2 Abs (T1D CD28). IL-6 (A), IL-8 **(B)**, and IL-17A **(C)** mRNA levels were measured by real-time PCR and values, normalized on GAPDH, expressed as arbitrary units. Median values: IL-6 (*n* = 10), DMSO = 0.77, DMSO CD28 = 18.99, ISA-2011B = 0.26, ISA-2011B CD28 = 8.27; IL-8 (*n* = 10), DMSO = 4, DMSO CD28 = 37.74, ISA-2011B = 4.75, ISA-2011B CD28 = 20.3; IL-17A (*n* = 6), DMSO = 1.18, DMSO CD28 = 72.42, ISA-2011B = 0.27, ISA-2011B CD28 = 10.32. Lines represent mean values and *P* values are indicated where significant (Mann–Whitney).

We have recently observed that the upregulation of pro-inflammatory cytokines and chemokines by CD28 was extremely relevant in inflammatory diseases, such as multiple sclerosis (MS), where we evidenced that CD28 stimulation induced a strong upregulation of pro-inflammatory cytokines in a class 1A PI3K-dependent manner (17). We extended this analysis to T lymphocytes from T1D. High levels of pro-inflammatory cytokines have been associated with diabetic vascular complications (32). Thus, we analyzed the extent of cytokine gene expression in primary T cells isolated from the peripheral blood of T1D patients. CD28 stimulation strongly increased the expression of IL-8, IL-6, and IL-17A. However, in contrast to that observed for MS patients (17), no significant differences in the entity of CD28 upregulation of cytokine gene expression were observed in T1D compared with HD (Figure S4 in Supplementary Material). Pre-treatment of cells with ISA-2011B significantly impaired CD28-induced upregulation of all cytokines (Figures 6A–C).

These data identify PIP5Kα as a potential therapeutic target to dampen CD28 pro-inflammatory signals in T1D T cells.

## Discussion

PIP2 is a pivotal regulator of TCR signaling, by serving as a precursor of important second messengers and by directly binding several signaling proteins regulating T cell activation (5, 33). During the early phases of TCR engagement by peptide–MHC complexes presented on the surface of APC, PIP2 concentrates at the IS (34) where it is rapidly metabolized by PLCγ1 for inducing sustained Ca^2+^ mobilization, IL-2 gene expression, and optimal T cell proliferation (35). CD28 amplifies and sustains TCR-dependent PLCγ1-mediated hydrolysis of PIP2 (36), thus leading to the generation of IP3 second messenger and the consequent release of Ca^2+^ from the intracellular stores and from the CRAC channels in the plasma membrane (37, 38). PIP5Kα isoform has been shown recruited to the plasma membrane in response to several receptors, where it synthesizes the PIP2 levels necessary for PLCγ activity, IP3 generation, and optimal Ca^2+^ influx (39–41). More recently, we evidenced that CD28 recruits and activates PIP5Kα at the interface between T cells and APC, thus strongly contributing to PIP2 synthesis (3, 4). Therefore, inhibiting the biosynthesis of PIP2 by targeting PIP5Kα may be useful to treat immune-based diseases, such as autoimmunity. Herewith, we demonstrate that PIP5Kα inhibitor, ISA-2011B, is effective in inhibiting the CD28 costimulatory signals essential for optimal TCR-induced Ca^2+^ influx, NF-AT transcriptional activation, and IL-2 gene expression.

A critical contribution of PIP2 to T cell biological functions is to serve as a substrate to class 1 PI3K. Class 1A PI3K phosphorylates PIP2 and generates PIP3 (42), which by binding the PH domains of several signaling molecules, such as phosphoinositide-dependent protein kinase 1 (PDK1), AKT, and Vav1 (43), strongly contributes to the activation of the NF-κB pathway (12–15). The transcription factors of Rel/NF-κB family are critical regulators of the immune system by inducing the expression of over 150 inflammatory cytokines and chemokines (44). CD28 is known to be the main activator of PI3K. Indeed, CD28 short cytoplasmic tail contains an N-terminal YMNM motif that following phosphorylation binds to the p85 subunit of class 1A PI3K (45–47), in a TCR-independent manner (48). Furthermore, CD28 also ensures the replenishment of PIP2 pools essential for optimal PI3K activity by recruiting and activating PIP5Kα through its C-terminal proline rich motif (3, 4). This autonomous capability of CD28 results to the selective activation of a non-canonical NF-κB2-like cascade leading to the expression of several genes, including survival genes of the Bcl-2 family (16), the LTR of HIV-1 virus (12), and pro-inflammatory cytokine/chemokine (17, 18). Our data on the inhibitory effects mediated by ISA-2011B on CD28-delivered signals regulating both NF-κB activation and pro-inflammatory cytokine/chemokine gene expression strongly support a role of PIP5Kα in regulating PI3K-dependent inflammatory functions of T lymphocytes.

The pro-inflammatory functions of CD28 may be particularly important in the context of inflammatory diseases. For instance, we have recently evidenced that CD28-mediated upregulation of pro-inflammatory cytokines (i.e., IL-8, IL-6, IL-21, and IL-17A) was higher in human primary T lymphocytes from relapsing-remitting multiple sclerosis patients (RRMS) compared with HD (17). We extended these data by demonstrating that CD28 autonomous signaling is active in upregulating pro-inflammatory cytokine/chemokine expression and Th17 amplification also in T1D. T1D is an autoimmune disease caused by the selective disruption of pancreatic islet cells by autoreactive T lymphocytes and to lifelong dependence on exogenous insulin therapy. The pathogenetic basis of T1D involves both genetic and environmental factors (49, 50). High plasma concentrations of pro-inflammatory cytokines have been detected in patients with T1D and are thought to be responsible for long-term micro and macrovascular diabetic complications (51, 52). Although chronic hyperglycemia has been considered the major determinant of onset and progression of endothelial alterations (32, 53), patients under insulin intensive therapy continue to suffer from vascular complications (54). Thus, identifying factors and/or molecules, which may contribute to sustain inflammation in T1D, may effectively be useful for treating or even preventing disease progression.

Herewith, we provide evidence that CD28 stimulation in the absence of TCR engagement may contribute in elevating the levels of pro-inflammatory cytokines in T1D patients. In particular, we found that CD28 stimulation strongly upregulated IL-6 and IL-17A cytokine gene expression in T lymphocytes from T1D. High levels of IL-6 (55, 56) and IL-17 (57–59) have been reported in T1D patients, thus underlying the emerging role of Th17 cell subset in the pathogenesis of T1D (60). Honkanen et al. showed that peripheral blood CD4^+^ T cells from new-onset T1D children produce higher levels of IL-17 (58). Similarly, Marwaha and colleagues found an increased number of CD4^+^ T cells that produce IL-17 in new-onset T1D children (59). The increased levels of IL-17 in T1D have been attributed to the presence of pro-inflammatory cytokines that drive toward Th17 differentiation (55). In particular, Th17 cell differentiation in humans is orchestrated by IL-6 (61). Recent data from Hundhausen et al. evidenced that IL-6 responsiveness is enhanced in T lymphocytes from T1D (62). Consistently, we found that CD28 stimulation strongly upregulated IL-6 and IL-17A expression in T cells from T1D patients. As observed in MS patients (17), CD28-mediated IL-17A gene expression was delayed (24 h) when compared with IL-6 (6 h), thus suggesting that CD28-induced production of IL-6 in turn cooperates with CD28 signaling in mediating IL-17A gene expression. These data are consistent with those of Santarlasci et al., who demonstrated that CD28 stimulation alone could trigger Th17 T cells to produce IL-17A in an NF-κB-dependent manner (63).

By using specific pharmacological inhibitors, we evidenced that CD28-induced upregulation of pro-inflammatory cytokines in MS patients was dependent on CD28-associated class 1A PI3K activation (17). Highly selective small molecule inhibitors of class 1A inhibitors are currently in pre-clinical and clinical trials for the treatment of several inflammatory and autoimmune diseases (64). Since PIP2 is an essential limiting factor ensuring that the activation of PI3K and PIP5Kα is the main regulator of CD28-mediated refilling of PIP2 pools (3, 33), PIP5Kα may represent a valid therapeutic candidate for treating inflammatory diseases. Our data on the strong inhibitory effect exerted by treating primary T cells with ISA-2011B suggest that targeting the CD28/PIP5Kα signaling pathway may, therefore, provide an attractive therapeutic approach for the resolution of injurious inflammatory processes in autoimmune diseases. Clearly a deeper characterization of the molecular pathways regulated by CD28 and PIP5Kα is required to open up new avenues to target PIP5Kα.

## Ethics Statement

This study was carried out in accordance with the recommendation of the Expert Committee on the Diagnosis and Classification of Diabetes Mellitus guidelines and Ethical Committee of the S.M. Goretti Hospital (Sapienza University, Rome Italy) with written informed consent from all subjects. All subjects gave written informed consent in accordance with the Declaration of Helsinki.

## Author Contributions

MK performed the research and data analyses; NC, MM, and CC performed parts of the research; FL and CM contributed clinical information and samples for the study; JP contributed with PIP5K inhibitor and commented on manuscript; RG helped in designing the study and in manuscript writing; RB contributed clinical information and samples for the study and commented on manuscript; LT designed the study, coordinated the work, and wrote the manuscript.

## Conflict of Interest Statement

The authors declare that the research was conducted in the absence of any commercial or financial relationships that could be construed as a potential conflict of interest.
