# Supplementary figures and images for: ISA-2011B, a Phosphatidylinositol 4-Phosphate 5-Kinase α Inhibitor, Impairs CD28-Dependent Costimulatory and Pro-inflammatory Signals in Human T Lymphocytes
Source: Front Immunol. 2017 Apr 26;8:502. doi: 10.3389/fimmu.2017.00502 (PMC5405084; doi:10.3389/fimmu.2017.00502)

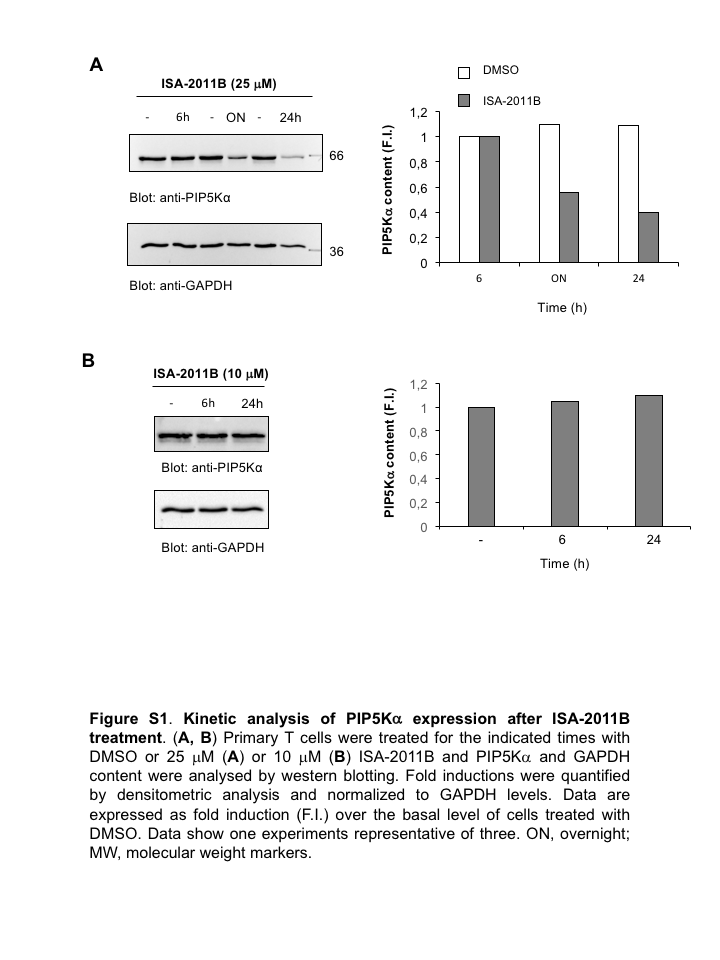

Supplement: Supplementary file 1 [file image_1.tif]

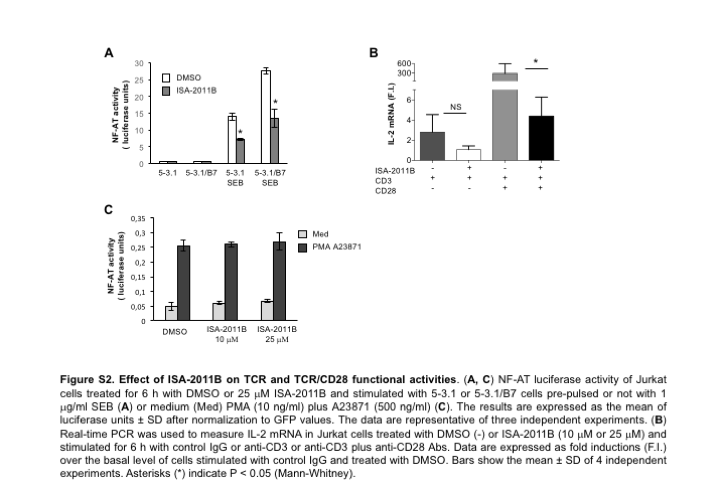

Supplement: Supplementary file 2 [file image_2.tif]

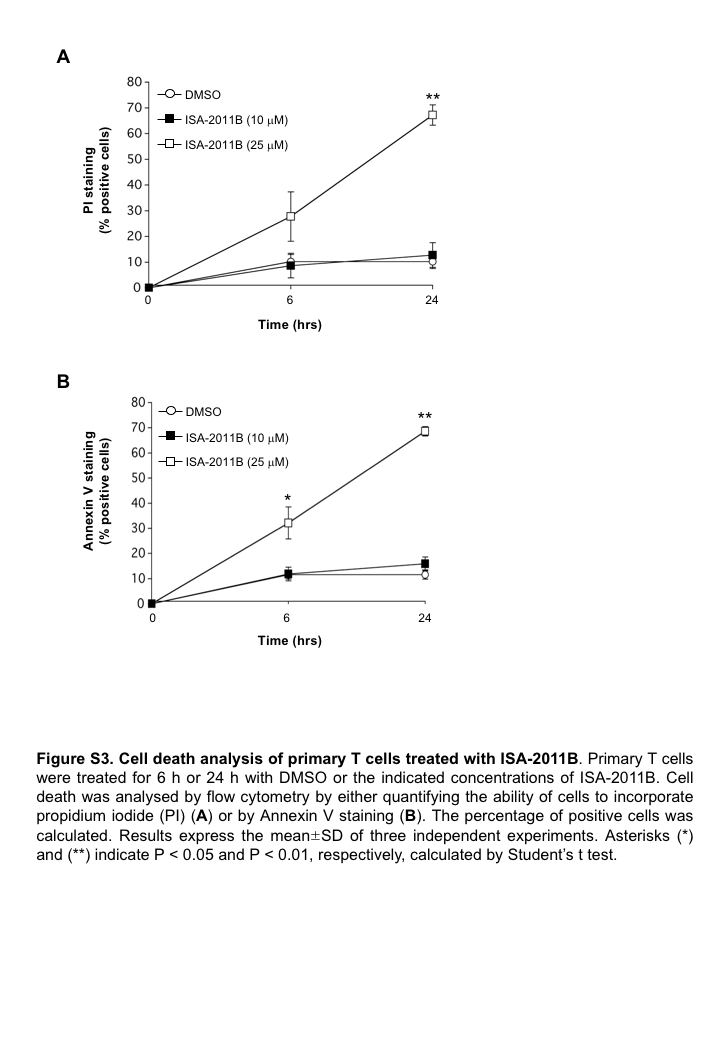

Supplement: Supplementary file 3 [file image_3.tif]

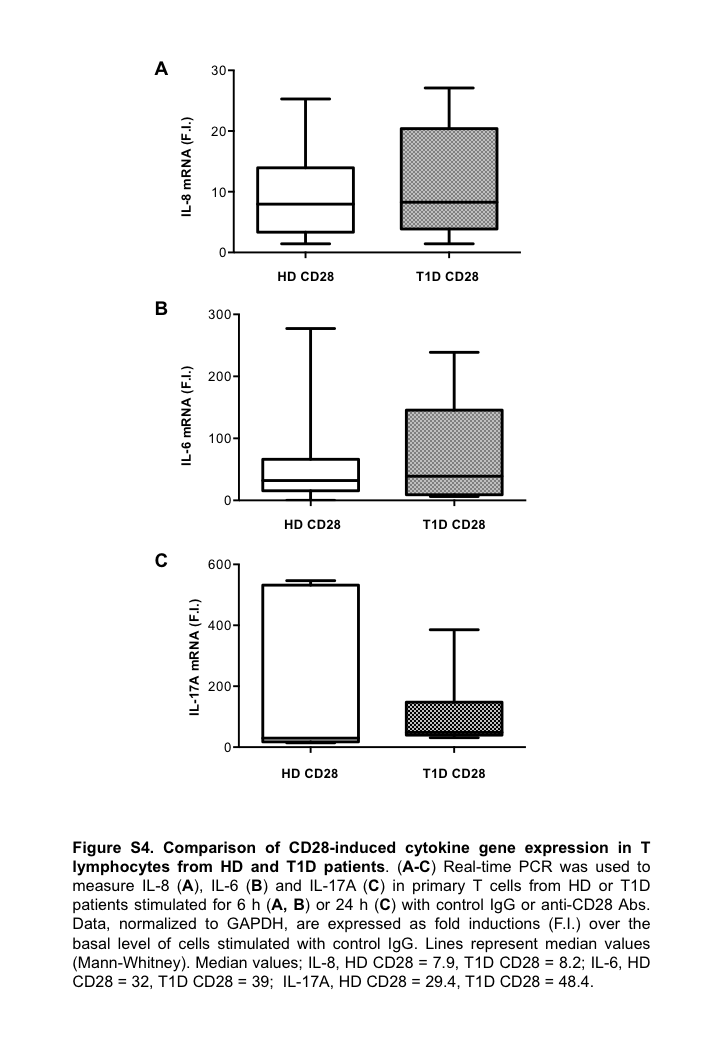

Supplement: Supplementary file 4 [file image_4.tif]
